# Accuracy of lung nodule density on HRCT: analysis by PSF‐based image simulation

**DOI:** 10.1120/jacmp.v13i6.3868

**Published:** 2012-11-08

**Authors:** Ken Ohno, Masaki Ohkubo, Janaka C Marasinghe, Kohei Murao, Toru Matsumoto, Shinichi Wada

**Affiliations:** ^1^ Department of Radiological Technology School of Health Sciences Faculty of Medicine Niigata University Niigata 951‐8518 Japan; ^2^ Fujitsu Ltd Tokyo 144‐8588 Japan; ^3^ Kensei Clinic Chiba 262‐0032 Japan

**Keywords:** point spread function (PSF), computed tomography (CT), lung cancer, high‐resolution computed tomography (HRCT), nodule density

## Abstract

A computed tomography (CT) image simulation technique based on the point spread function (PSF) was applied to analyze the accuracy of CT‐based clinical evaluations of lung nodule density. The PSF of the CT system was measured and used to perform the lung nodule image simulation. Then, the simulated image was resampled at intervals equal to the pixel size and the slice interval found in clinical high‐resolution CT (HRCT) images. On those images, the nodule density was measured by placing a region of interest (ROI) commonly used for routine clinical practice, and comparing the measured value with the true value (a known density of object function used in the image simulation). It was quantitatively determined that the measured nodule density depended on the nodule diameter and the image reconstruction parameters (kernel and slice thickness). In addition, the measured density fluctuated, depending on the offset between the nodule center and the image voxel center. This fluctuation was reduced by decreasing the slice interval (i.e., with the use of overlapping reconstruction), leading to a stable density evaluation. Our proposed method of PSF‐based image simulation accompanied with resampling enables a quantitative analysis of the accuracy of CT‐based evaluations of lung nodule density. These results could potentially reveal clinical misreadings in diagnosis, and lead to more accurate and precise density evaluations. They would also be of value for determining the optimum scan and reconstruction parameters, such as image reconstruction kernels and slice thicknesses/intervals.

PACS numbers: 87.57.‐s, 87.57.cf, 87.57.Q‐

## I. INTRODUCTION

Lung cancer screening with low dose computed tomography (CT) was shown to be effective for the reduction of lung cancer mortality by the National Lung Screening Trial.^(1)^ The wide dissemination of high quality screening with multidetector‐row CT (MDCT) may further reduce the mortality due to lung cancer. When high‐resolution CT (HRCT) was applied to the lung nodules detected by CT screening, a quantitative evaluation of nodule size and density may be valuable.^2^, ^5^ The accuracy of such a quantitative analysis may also lead to clinically useful information. For accuracy analyses, many studies employ a phantom with artificial nodules,^6^, ^10^ which has the limitation that numerous nodules must be fabricated accurately in a variety of sizes and densities.

**Figure 1:**
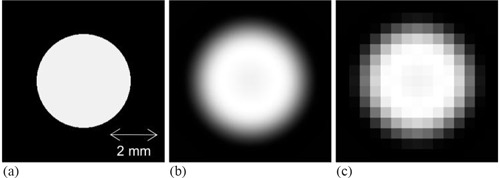
Generation of a simulated nodule: (a) object function obtained by assuming a pulmonary nodule; (b) the simulated blurred image obtained from image (a); (c) the image obtained by the resampling of image (b).

Prior studies have used computer simulation of CT images based on the spatial resolution of a CT system to investigate the accuracy of size and density measurements. The object functions used in those image simulations were designed to emulate cortical bone,^11^, ^12^ small high‐density structures (calcifications and stented vessels),^(13)^ and lung nodules.^(14)^ To the best of our knowledge, such simulated images were computed with a fine digital sampling pitch (the interval of the discrete data); one such example is illustrated in Fig. 1. In this approach, an arbitrary object function is numerically generated with a fine digital sampling pitch ((Fig. 1a). From the object function, the simulated blurred image is then computed ((Fig. 1b). This simulated image has the advantage of having an exact dependency on the characteristics of the spatial resolution of the CT system.^14^, ^17^ However, the image has a fine interval of the discrete data; therefore, we propose an additional resampling to obtain the clinical CT image such as the image indicated in (Fig. 1c).

In the present study, we measured the point spread function (PSF) in a CT system, and performed a lung nodule image simulation based on the PSF. Then, the simulated image was resampled at intervals equal to the pixel size and the slice interval found in clinical HRCT images. On the resulting virtual nodule image, we measured the nodule density by placing a region of interest (ROI) that is commonly used for routine clinical practice, and compared the measured value with the true value (a known density of object function). The accuracy of the density measurement was then quantitatively evaluated.

## II. MATERIALS AND METHODS

### A. Simulation of 3D CT image blurring

CT image blurring can be described by the PSF of the imaging system.^18^, ^19^ It can be assumed that a PSF is separable into a two‐dimensional (2D) PSF in the xy scan plane and a slice sensitivity profile (SSP) in the z direction perpendicular to the scan plane.^12^, ^14^ Then, the three‐dimensional (3D) CT image I(x, y, z) can be expressed as follows:

(1) I(x,y,z)=(O(x,y,z)∗∗PSF(x,y))∗SSP(z)

where *O*(*x, y z*) is the object function, *PSF*(*x, y*) and *SSP*(*z*) are the 2D PSF in the scan plane and SSP in the z‐axis, respectively; ** and * are the 2D and one‐dimensional convolutions, respectively.

In the current study, we measured the PSFs and SSPs in a CT scanner, in which the PSFs were obtained for three types of reconstruction kernels (FC10, FC50, and FC52). The SSPs were obtained for slice thicknesses of 1.0 and 2.0 mm, as described later. Object functions O(x, y, z) were numerically generated as ideal spheres with uniform density, by assuming solitary pulmonary nodules. The object functions of the spheres had a constant CT value of ‐400 Hounsfield Unit (HU)^(20)^ with various diameters ranging from 1.0 to 8.0 mm (with an increment of 0.25 mm). The CT value in the background of the spheres that were used to simulate the lung field was ‐900 HU. By Eq. (1), we computed the CT image I(x, y, z) from the object functions, using the measured PSFs and SSPs.

Several studies^12^, ^14^ have performed the above computer simulations. In those simulations, the PSFs, SSPs, and object functions were treated as discrete (digital) data; however, the interval of the discrete data was not specified. The interval should be set to be sufficiently fine so as to prevent the problem of aliasing. As an example, images are shown in (Figs. 1a) (O(x, y, z)) and 1(b) (I(x, y, z)), in which the interval of the discrete data was 22.9 μm on three axes (x, y, z); this value corresponded to the pixel size (22.9×22.9 μm) and the slice interval (22.9 μm) of the CT images. In the current study, we generated the object functions of spheres with discrete data intervals less than one‐fiftieth of the sphere diameter. Taking into consideration the limitations in available computer memory, the intervals ranged from 15.0 to 40.0 μm. Next, the measured PSFs and SSPs were resampled with the same data intervals as the object functions by using a linear interpolation, followed by the calculation in Eq. (1).

### B. Resampling of I(x, y, z) based on the pixel size and slice interval in a clinical HRCT image

The CT image I(x, y, z) was resampled by the standard digital sampling method, with intervals equal to the pixel size and slice interval found in clinical HRCT images. By this resampling, we transformed simulated blurred images into practical images that could be used for a clinical evaluation. We assumed the HRCT images were obtained by the targeted reconstruction for one side of the lung, with a field of view (FOV) of 200 mm (512×512 matrix), and with the same slice interval as the slice thickness (1.0 or 2.0 mm).^(25)^ Then, I(x, y, z) was resampled by linear interpolation in the xy plane with the interval of the pixel size (approximately 0.4 mm) on the x‐ and y‐axis, and on the z‐axis with the slice interval corresponding to the slice thickness (1.0 or 2.0 mm). In this work, the image obtained by the resampling operation is defined as '$I_{d}$', and is a 3D CT image volume data consisting of axial images. As an example, one axial slice from $I_{d}$ is shown in (Fig. 1c), which was obtained by resampling I(x, y, z) ((Fig. 1b) with intervals of 0.4, 0.4, and 1.0 mm on the x‐, y‐ and z‐axis, respectively.

The interval used for the resampling of I(x, y, z) on the x‐ and y‐axis was constant (i.e., the pixel size in a 200 mm FOV), while the interval on the z‐axis was changed from 50% to 100% of the slice thickness with an increment of 10%. Then, in resampling I(x, y, z) computed for a slice thickness of 1.0 mm, the intervals on the z‐axis were 0.5, 0.6, 0.7, 0.8, 0.9, and 1.0 mm, and in resampling I(x, y, z) computed for a slice thickness of 2.0 mm, the intervals were 1.0, 1.2, 1.4, 1.6, 1.8, and 2.0 mm. By resampling on the z‐axis with intervals that were less than 100% of the slice thickness, overlapping slices were generated; this was based on the assumption of overlapping image reconstruction with a slice interval less than the slice thickness. Various $I_{d}$ were obtained from one I(x, y, z) with changing slice intervals. We then expressed $I_{d}$ with subscripts describing the slice thickness and slice interval, as in ‘$I_{d(\text{slice}\;\text{thickness},\;\text{slice}\;\text{interval})}$’. For example, when I(x, y, z) computed for the slice thickness of 2.0 mm was resampled with a z‐axis interval of 1.4 mm, the obtained $I_{d}$ was expressed as $I_{d(2.0,\;1.4)}$.

### C. Offset between nodule center and image voxel center

**Figure 2:**
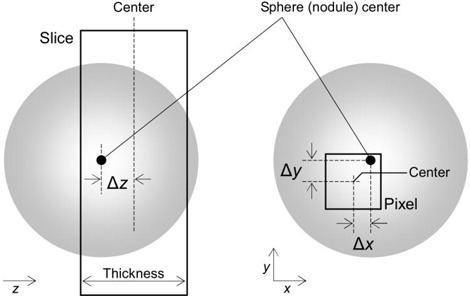
The offset (Δx, Δy and Δz) between nodule center and image voxel center are represented schematically. The voxel is the 3D region defined by the slice and pixel bounds.

In $I_{d}$, there was one voxel centered at the location nearest to the center of the sphere simulating a 3D nodule. The 3D offset of the voxel center relative to the sphere center is depicted schematically in Fig. 2, where the voxel represents a 3D region defined by the slice and pixel bounds. The offset was evaluated by Δx, Δy and Δz, as shown in Fig. 2. The values of Δx, Δy and Δz could lie within the following ranges:

(2) $$0\leq \Delta x\leq \frac{1}{2}\;pixel\;size$$

(3) $$0\leq \Delta y\leq \frac{1}{2}\;pixel\;size$$

(4) $$0\leq \Delta z\leq \frac{1}{2}\;slice\;thickness$$

where Eq. (4) is valid when the slice thickness is the same as the slice interval. When overlapping image reconstruction was performed with the slice interval set to be less than the slice thickness, the values of Δz could lie within the following range:

(5) $$0\leq \Delta z\leq \frac{1}{2}\;slice\;interval$$

The values of Δx, Δy and Δz in the $I_{d}$ depended on the sampling point locations in the resampling of I(x, y, z). The various $I_{d}$ were obtained by changing the sampling point locations so that the values of Δx, Δy and Δz changed from the minimum to the maximum values, as given by Eqs. (2), (3) and (5), respectively. The Δx and Δy parameters were changed by an increment of one‐fourth of the pixel size (an approximately 0.1 mm increment). The Δz parameter was changed by an increment of one‐tenth of the slice interval in resampling I(x, y, z) computed for the slice thickness of 1.0 mm; and was changed by an increment of one‐twentieth of the slice interval in resampling I(x, y, z) computed for the slice thickness of 2.0 mm.

Thus, we obtained various $I_{d}$ with variable offsets between the nodule center and voxel center (Δx, Δy and Δz), and also with the variable slice intervals described above. This offset value is an unknown factor in clinical CT scans.

### D. Validation of the resampling

A phantom experiment was performed to validate the method of resampling I(x, y, z) with the consideration of the offset between nodule center and image voxel center, mentioned in Materials & Methods Sections B and C above. We used a commercially available phantom (high‐contrast CT test phantom; MHT‐type, Kyoto Kagaku Co., Ltd., Kyoto, Japan) which was filled with lung tissue‐equivalent material and had a section including five uniform spherical objects (diameters of 2, 3, 5, 7, and 10 mm) made of soft tissue‐equivalent material.

We scanned the section in the phantom using the same scanner and conditions detailed later for the measurements of PSF and SSP (see Materials & Methods Section G below). The image reconstruction was performed using the FC10, FC50, and FC52 kernels, slice thicknesses of 1.0 and 2.0 mm with an interval of 0.1 mm, and a FOV of 50 mm. The obtained images had a small interval of the discrete data (i.e., approximately 0.1 mm pixel size and 0.1 mm slice interval). Then, in the same manner as described above, those images were resampled in the xy scan plane with the interval equal to the pixel size in the 200 mm FOV images, and were resampled on the z‐axis with the interval corresponding to the slice thickness. This resampling was done by assuming two cases of minimum and maximum values of nodule center offset (Δx, Δy and Δz in Eqs. (2), (3), and (5)).

Next, the phantom was further scanned after changing the reconstruction FOV to 200 mm (other scan/reconstruction parameters were not changed); at this time, the scanning was done repeatedly while varying the phantom location in the xy plane to experimentally demonstrate the effect of nodule center offset. The theoretical values of Δx and Δy ranged from 0.0 to approximately 0.2 mm (see Eqs. (2) and (3)). The changing of phantom location was done manually by adjusting a mechanical apparatus used for supporting the phantom in the CT gantry; however, the procedure for moving the phantom does not have accuracy less than 0.1 mm. Therefore, the scanning was repeated while changing the phantom location in each x and y direction by the smallest possible variation of less than 0.1 mm; a total of 36 scans (in six x and y locations, respectively) were done in this way. On each image obtained by these scans, we measured the maximum pixel value (CT value) around the approximate object center. When investigating the obtained values of all images, we chose one image having the maximum value and another image having the minimum value, because it was assumed that the image with the maximum value had the minimum Δx and Δy, while the image with the minimum value had the maximum Δx and Δy. This investigation was repeated for each object (diameters of 2–10 mm). Also, with regard to Δz, by investigating the object size and density on all images reconstructed at various slice locations, we chose one image beforehand that seemed to have a larger size and higher density; this image was assumed to have the minimum Δz. Then, based on the slice location of this image, we could choose another image that had the maximum Δz (see Eq. (5)). For preliminary verification of the scans of the phantom and the image choice, the location of the object (2 mm diameter sphere) on the image chosen as having the maximum Δx and Δy was compared with the location on the image chosen as having the minimum Δx and Δy. The distance between these locations was measured on additional images obtained by targeted reconstruction with a 50 mm FOV, and using the CT system's built‐in tools such as the zoom and distance measure. The distance was found to be approximately 0.2 mm in both the x and y directions; this value was equivalent to the above ideal value of maximum Δx and Δy (i.e., one half of the pixel size of the image with 200 mm FOV), and suggested successful validation of the two obtained images.

The image obtained from the 50 mm FOV image by the resampling was compared with corresponding 200 mm FOV image, for two cases of minimum and maximum values of Δx, Δy and Δz. By this comparison, we confirmed the validity of the resampling that took into consideration the nodule center offset.

### E. Accuracy of lung nodule density evaluation

**Figure 3:**
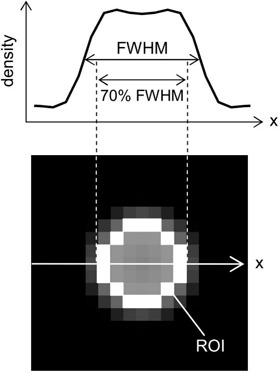
The region of interest (ROI) used for measurement of nodule density: (lower) the full width at half maximum (FWHM) was estimated from the density profile (upper) along a horizontal line (indicated by a line in the image) through the sphere center. The 70% of FWHM was applied to the diameter of the circular ROI.

In $I_{d}$, there was one axial image that was centered at a location nearest to the center of the sphere that simulated a 3D nodule. Using this image slice, we measured the nodule density. As a ROI is commonly used for clinical measurements of nodule density, we employed a circular ROI, as shown in Fig. 3. The full width at half maximum (FWHM) was estimated from the density profile measured through the sphere center. The 70% of FWHM was applied to the diameter of the circular ROI. The mean density in the ROI was then selected as the measured nodule density. As described above, the object functions had a constant CT value of ‐400 HU; this CT value was taken to be the true density. The measured nodule density was then compared with the true density to investigate the accuracy of clinical density evaluations.

### F. Addition of simulated nodules (Id) to lung CT image

**Figure 4:**
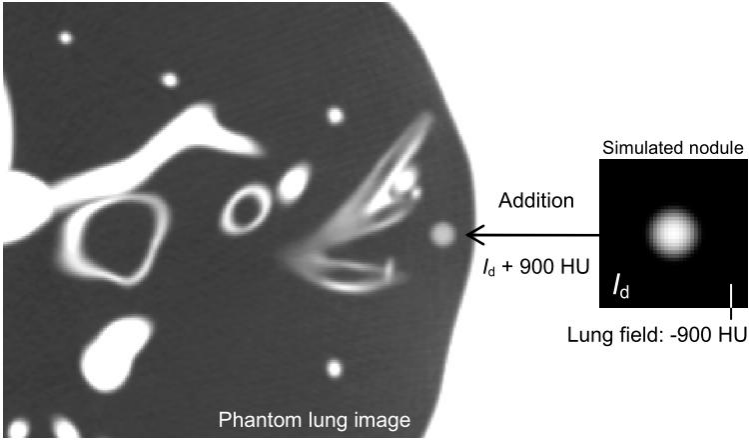
Addition of a simulated nodule image to the phantom lung image. The simulated nodule image $I_{d}$ (right) was added by the constant of 900 HU; then, it was added to the lung field in the phantom image (left).

In $I_{d}$, there was one axial image that was centered at a location nearest to the center of the sphere; this image was used to generate a virtual nodule on the practical lung image. The simulated nodule on the image was then added to the lung image, as illustrated in Fig. 4. We obtained the lung image using a phantom, described in more detail in the next Section. The background region in the simulated nodule image had a density of ‐900 HU, as described above for the object function setting. By adding 900 HU to the simulated image, the CT value of the background region was set to 0 HU. Then we selected a region in the phantom image that was free of anatomic structures, such as the bronchus or blood vessels. At this image region, the simulated image was added to the phantom lung image, as indicated in Fig. 4. By this addition, we generated virtual nodules, thus enabling a qualitative assessment of the nodules based on the practical CT image.

### G. Equipment and imaging parameters

A four‐detector‐row CT scanner (Asteion; Toshiba Medical Systems, Tokyo, Japan) was used for imaging. The PSFs were measured in the scanner by a recently proposed method that can be used to determine the 2D PSF in the scan plane with one scan of the phantom described above (Materials & Methods Section D), accompanied by verification.^16^, ^17^ Another section of the phantom including five cylindrical objects (diameter of 2, 3, 5, 7, and 10 mm) was used. The phantom was imaged in the CT scanner using a tube voltage of 120 kV and current of 200 mA, and the targeted reconstructions were performed using multiple reconstruction kernels with a FOV of 100 mm, and a matrix size of 512×512. We chose three types of reconstruction kernels in the scanner: FC10 (standard abdominal imaging), FC50 (standard lung imaging), and FC52 (high‐resolution lung imaging). Then, three types of PSFs were obtained for the three reconstruction kernels.

For the measurement of SSPs, we used a Gold Disk Delta phantom (Kyoto Kagaku Co., Ltd., Kyoto, Japan) consisting of a 50 μm thick gold disk of 1 mm diameter placed in a tissue equivalent material (acrylic). The phantom was imaged using settings of 120 k V, 200 mA, 4×1 mm collimation, 1.5 s/rot, and a pitch factor of 0.75. The image reconstructions were performed using slice thicknesses of 1.0 and 2.0 mm with intervals of 0.1 mm. This produced two types of SSPs for two different slice thicknesses.

The phantom used for the addition of simulated nodules, as described previously, was a one‐piece anthropomorphic torso phantom (CT Torso phantom; CTU‐41, Kyoto Kagaku Co., Ltd., Kyoto, Japan), which contained anatomical structures (Fig. 4). Each simulated organ in the phantom possesses a particular CT number corresponding to the actual organ in the human body. The chest region in the phantom was imaged using settings of 120 kV, 200 mA, 4×1 mm collimation, 1.5 s/rot, and a pitch factor of 0.75. The image reconstruction was performed using the FC52 kernel and a slice thickness of 2.0 mm. The targeted reconstruction was performed with a FOV of 200 mm.

All calculations were carried out using the technical computing software MATLAB (The MathWorks Inc., Natick, MA).

## III. RESULTS

### A. Simulated nodule image (Id)

**Figure 5:**
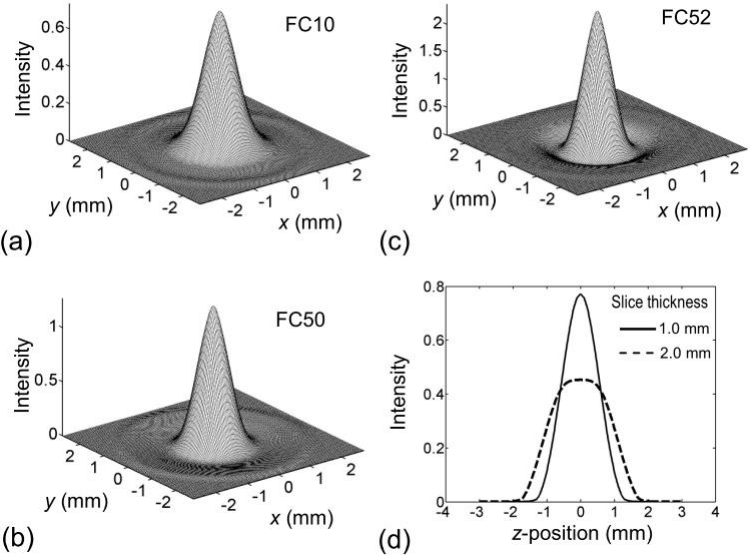
The point spread functions (PSF) measured for three types of reconstruction kernels, FC10 (a), FC50 (b), and FC52 (c). SSPs measured for two reconstruction slice thicknesses 1.0 and 2.0 mm (d).

The measured PSFs and SSPs are shown in Fig. 5. Three types of PSFs were obtained for three reconstruction kernels of FC10, FC50, and FC52. Two SSPs were obtained for slice thicknesses of 1.0 and 2.0 mm. In this study, all numerical simulations of the 3D image blurring based on Eq. (1) were accomplished using these PSFs and SSPs.

**Figure 6:**
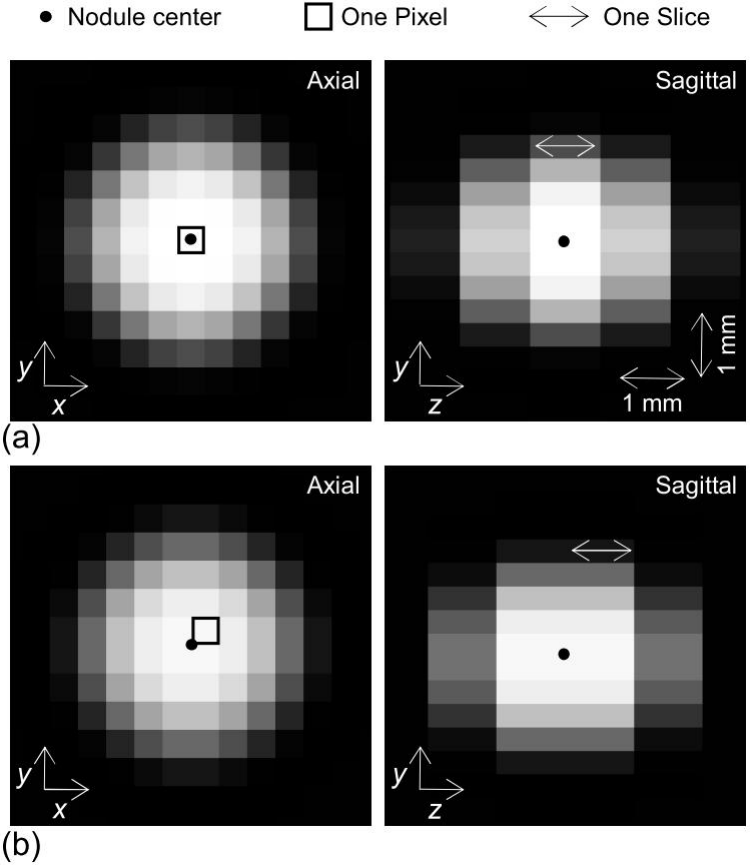
Two cases of the 3D offset between nodule center and voxel center. The images (a) (left; axial image, right; sagittal image) were obtained with the minimum offset, while the images (b) were obtained with the maximum offset. Dots show the spatial location of the nodule center. One pixel and one slice that were centered at a location nearest to the nodule center are indicated by square and double arrow, respectively.

The 3D CT image volume data $\left(I_{d}\right)$ was obtained by resampling I(x, y, z) computed by Eq. (1). Some slices in $I_{d}$ are shown in Fig. 6; $I_{d}$ was obtained by assuming a sphere (object function) diameter of 3.0 mm, the FC50 reconstruction kernel, a slice thickness of 1.0 mm, and a slice interval of 1.0 mm (i.e., such that $I_{d}$ was expressed as $I_{d(1.0,\;1.0)}$ according to $I_{d(\text{slice}\;\text{thinkness},\;\text{slice}\;\text{interval})}$, as defined previously. The images in (Fig. 6a) were obtained with the minimum offset between the sphere center and voxel center (see Eqs. (2)–(5)), that is, the values of Δx, Δy and Δz were zero, while the images in (Fig. 6b) were obtained with the maximum offset. The simulated nodule varied with the offset.

### B. Validation of the resampling

**Figure 7:**
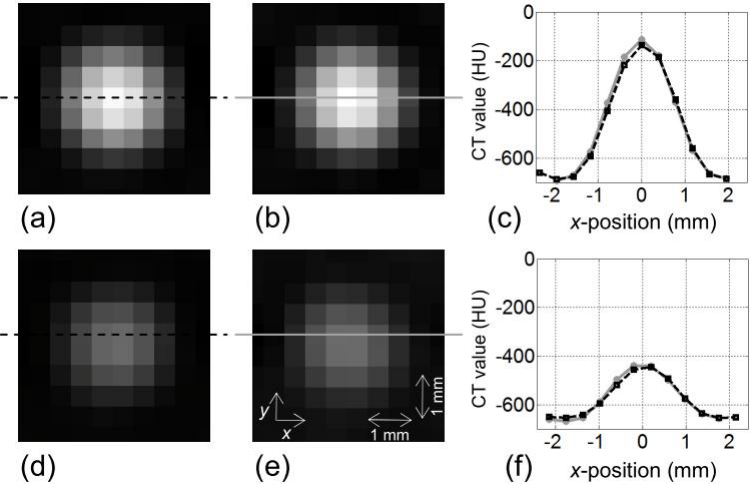
Comparison of resampled image and measured image, for the spherical object with diameter of 2 mm: (a) image obtained from the 50 mm FOV image by the resampling with the minimum values of Δx, Δy and Δz; (b) image with the 200 mm FOV; (c) CT value profile for 7(a) (dashed‐line) and for 7(b) (gray‐line). These results were for the slice thickness of 2.0 mm and reconstruction kernel of FC50. Similar results (d–f) for the maximum values of Δx, Δy and Δz.

The image obtained from the 50 mm FOV image by the resampling with the minimum values of Δx, Δy and Δz ((Fig. 7a) was compared with the corresponding 200 mm FOV image ((Fig. 7b) that was chosen from images obtained by scanning the phantom while varying its location in the CT gantry (see Materials & Methods Section D). They showed good agreement; this was also confirmed by a comparison of their respective CT value profiles ((Fig. 7c). In the same manner, the comparison was done for the case with the maximum values of Δx, Δy and Δz ((Figs. 7d–f)); again, a good agreement was found. We also obtained similarly good results for other objects with various diameters (3, 5, 7, and 10 mm) and for other reconstruction parameters, such as slice thickness (1.0 mm) and kernels (FC10 and FC52) (results not shown).

### C. Lung nodule density evaluation

**Figure 8:**
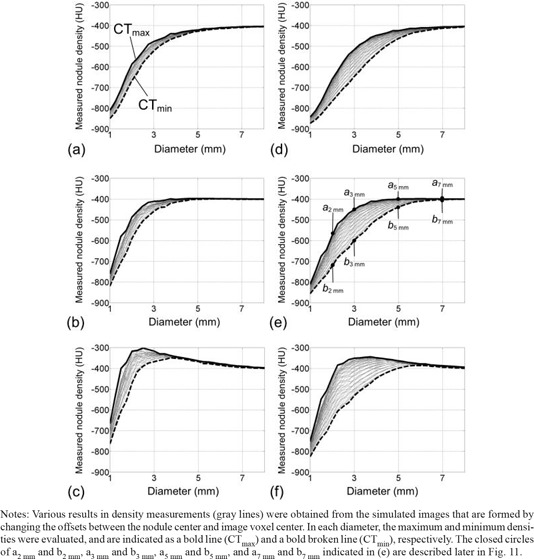
The measured densities in simulated nodule images, for diameters from 1.0 to 8.0 mm for: slice thicknesses of 1.0 mm (a–c) and for 2.0 mm (d–f), and reconstruction kernels of FC10 (a, d), FC50 (b, e) and FC52 (c, f).

Notes: Various results in density measurements (gray lines) were obtained from the simulated images that are formed by changing the offsets between the nodule center and image voxel center. In each diameter, the maximum and minimum densities were evaluated, and are indicated as a bold line $\left({\text{CT}}_{\max}\right)$ and a bold broken line $\left({\text{CT}}_{\min}\right)$, respectively. The closed circles of $a_{2\;\text{mm}}$ and $b_{2\;\text{mm}}$, $a_{3\;\text{mm}}$ and $b_{3\;\text{mm}}$, $a_{5\;\text{mm}}$ and $b_{5\;\text{mm}}$, and $a_{7\;\text{mm}}$ and $b_{7\;\text{mm}}$ indicated in (e) are described later in Fig. 11.

Using the results of $I_{d}$, the nodule densities were evaluated (Fig. 8). (Figure 8a) shows the measured nodule density in $I_{d(1.0,\;1.0)}$ obtained for the FC10 kernel. The measured density that is changing with the diameter is regarded as one data series, and is drawn as a gray curved line in the figure. In this process, we changed the offset between the sphere center and image voxel center, and obtained many data series, depicted as many gray curved lines in (Fig. 8a). Using these density curves, the maximum density in each diameter was obtained and regarded as one data series. We defined this curve as '${\text{CT}}_{\text{max}}$‘ and it is shown in (Fig. 8a) as a bold line. Also, the minimum density in each diameter was obtained, which was expressed in (Fig. 8a) as a bold broken line; we defined this curve as '${\text{CT}}_{\text{min}}$‘. In the same way, the results for the slice thicknesses of 1.0 and 2.0 mm and for the reconstruction kernels FC10, FC50, and FC52 are shown in Fig. 8. We compared the measured nodule density with the true value (‐400 HU) of the object. For FC10 and FC50, when the true diameter was decreased, the measured density was underestimated; when the true diameter was increased, the measured density was closer to the true value. For FC52, when the true diameter was increased from the minimum value, the measured density varied from being underestimated to being overestimated, and then was closer to the true value.

**Figure 11:**
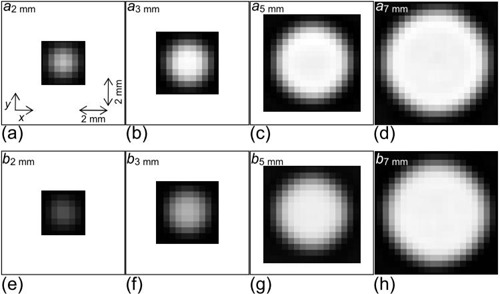
Phantom sphere images (from left to right, diameters of 2, 3, 5, and 7 mm) obtained experimentally (200 mm FOV): (a–d) in the case of the minimum offset between sphere center and voxel center; (e–h) in the case of the maximum offset. All images are displayed with the same window and level settings.

In Fig. 8, for each diameter, the measured nodule density was found to have the potential to fluctuate from ${\text{CT}}_{\text{min}}$ to ${\text{CT}}_{\text{max}}$, depending on the offset; the fluctuation in nodule density measurement when the slice thickness was 2.0 mm tended to be bigger than when the slice thickness was 1.0 mm. The fluctuation also tended to increase according to the following order of the reconstruction kernel: FC52, FC50, and FC10.

**Figure 9:**
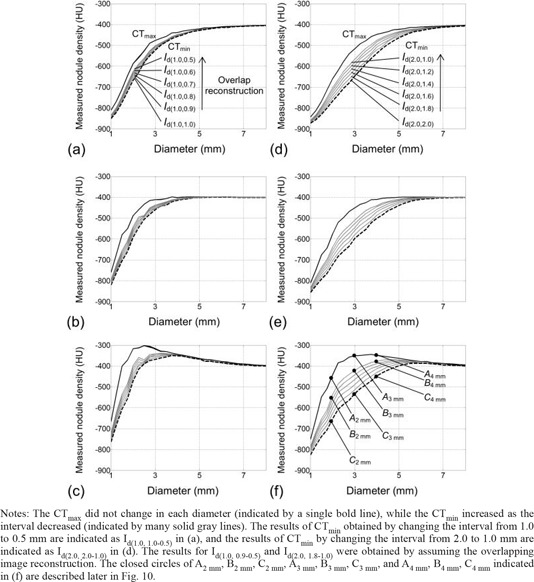
The results of the ${\text{CT}}_{\text{max}}$ and ${\text{CT}}_{\text{min}}$ (defined in obtained by changing the slice interval for: slice thickness of 1.0 mm (a–c), and for slice thickness of 2.0 mm (d–f), and reconstruction kernels of FC10 (a, d), FC50 (b, e), and FC52 (c, f).

Notes: The ${\text{CT}}_{\text{max}}$ did not change in each diameter (indicated by a single bold line), while the ${\text{CT}}_{\text{min}}$ increased as the interval decreased (indicated by many solid gray lines). The results of ${\text{CT}}_{\text{min}}$ obtained by changing the interval from 1.0 to 0.5 mm are indicated as $I_{d(1.0,\;1.0‐0.5)}$ in (a), and the results of ${\text{CT}}_{\text{min}}$ by changing the interval from 2.0 to 1.0 mm are indicated as $I_{d(2.0,\;2.0‐1.0)}$ in (d). The results for $I_{d(1.0,\;0.9‐0.5)}$ and $I_{d(2.0,\;1.8‐1.0)}$ were obtained by assuming the overlapping image reconstruction. The closed circles of $A_{2\;\text{mm}}$, $B_{2\;\text{mm}}$, $C_{2\;\text{mm}}$, $A_{3\;\text{mm}}$, $B_{3\;\text{mm}}$, $C_{3\;\text{mm}}$, and $A_{4\;\text{mm}}$, $B_{4\;\text{mm}}$, $C_{4\;\text{mm}}$ indicated in (f) are described later in Fig. 10.

We changed the interval used for the resampling of I(x, y, z) on the z‐axis. This interval corresponded to the slice interval, and the interval that was less than the slice thickness was used for the overlapping reconstruction. For each interval, we obtained the result corresponding to Fig. 8, and ${\text{CT}}_{\text{max}}$ and ${\text{CT}}_{\text{min}}$ were determined for each result in the same way. The data (${\text{CT}}_{\text{max}}$ and ${\text{CT}}_{\text{min}}$) for intervals from 50% to 100% of the slice thickness are all shown in Fig. 9. The results of ${\text{CT}}_{\text{max}}$ did not differ from the results in Fig. 8 when changing the interval, while the ${\text{CT}}_{\text{min}}$ increased as the interval decreased (as indicated in Fig. 9 as gray solid lines). The increase of ${\text{CT}}_{\text{min}}$ corresponded to the decrease of the fluctuation from ${\text{CT}}_{\text{min}}$ to ${\text{CT}}_{\text{max}}$ in the nodule density measurement; this decrease of fluctuation resulted in a stable measurement of nodule density with good reproducibility. The decrease in fluctuation for a slice thickness of 2.0 mm tended to be relatively larger than for a slice thickness of 1.0 mm, and tended to be relatively large in the following order of the reconstruction kernel: FC52, FC50, and FC10.

**Figure 10:**
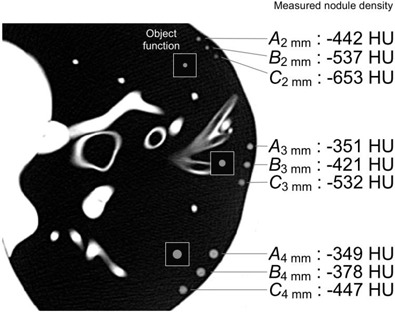
Simulated nodules that were used to obtain the results indicated as A–C for 2, 3, and 4 mm diameters in were added to the phantom lung image. The object functions are also shown. The density value of each nodule A–C is shown, which are measured with the ROI described in Fig. 3.

### D. Qualitative assessment of simulated nodules (Id) based on the practical CT image

For the qualitative assessment of nodule density, simulated nodule images were added to the phantom lung image (shown in Fig. 10). The simulated nodule images had previously been used for the determination of the data indicated as A, B, and C (for 2, 3, and 4 mm diameters) in (Fig. 9f). The object functions are also shown, in which density is constant but the diameter is 2, 3, and 4 mm. Regardless of the same setting of density (‐400 HU), the density of the virtual nodules $C_{2\;\text{mm}}$ and $C_{3\;\text{mm}}$ in Fig. 10 was observed to be lower than in nodules $A_{2\;\text{mm}}$ and $A_{3\;\text{mm}}$, respectively. This difference in density was caused by the offset between the sphere center and voxel center. By contrast, the densities of nodules $B_{2\;\text{mm}}$ and $B_{3\;\text{mm}}$ were observed to be similar to nodules $A_{2\;\text{mm}}$ and $A_{3\;\text{mm}}$, respectively. However, the densities of $A_{4\;\text{mm}}$, $B_{4\;\text{mm}}$, and $C_{4\;\text{mm}}$ were observed to be similar. The difference in density values between nodules $A_{2\;\text{mm}}$ and $C_{2\;\text{mm}}$, and the difference between $A_{3\;\text{mm}}$ and $C_{3\;\text{mm}}$ were approximately 210 and 180 HU, respectively. These differences suggest that the fluctuation in density measurements of small nodules may occur in clinical practice.

## IV. DISCUSSION

To enable an accuracy analysis for clinical evaluation of lung nodule density, we simulated nodule images based on the spatial resolution of the CT system, and resampled the simulated images by intervals equal to the pixel size and the slice interval found in clinical HRCT images. In this resampling, the offset between the nodule center and image voxel center was taken into consideration. To the best of our knowledge, this approach has not been performed in previous simulations based on spatial resolution,^11^, ^14^ and its validity was verified by a phantom experiment (Fig. 7). The accuracy of lung nodule density evaluations was quantitatively demonstrated (Figs. 8 and 9) by including practical effects such as the offset between the nodule center and voxel center, and slice interval.

In addition, in Fig. 11, we show images of spheres in the phantom which were obtained experimentally by a validation study described in the Materials & Methods Section D. The phantom spheres had a constant density and diameters of 2, 3, 5, and 7 mm; therefore, by using those images, we could verify the relative difference in density data indicated as $a_{2,3,5,7\;\text{mm}}$ and $b_{2,3,5,7\;\text{mm}}$ in (Fig. 8e). For example, the differences between the density of spheres in (Fig. 11a) and (e), and between (Fig. 11b) and (f) were observed to be larger than those between (Fig. 11c) and (g). The density of spheres in (Fig. 11d) and (h) were similar (i.e., they converged to the true density). Furthermore, the density in (Fig. 11b) seemed to be similar to that in (Fig. 11g). These findings agreed well with results shown in (Fig. 8e), suggesting the validity of results in Fig. 8.

The accuracy of density measurement in clinical practice could be estimated by the results shown in Fig. 8. For example, for the FC50 reconstruction kernel and a slice thickness of 1.0 mm ((Fig. 8b), accurate and precise measurement of density was made as the nodule diameter was increased by more than approximately 5 mm. Meanwhile, for a slice thickness of 2.0 mm ((Fig. 8e), accurate and precise measurement of density was made as the nodule diameter was increased by more than approximately 7 mm. The fluctuation in measured density was found to be noticeable when using a slice thickness of 2.0 mm. By our proposed method, we can analyze the fluctuations caused by the change in the offset between the nodule center and voxel center, which is an unknown factor in clinical CT scans. This fluctuation could be reduced by an overlap reconstruction (Fig. 9). These results may be useful not only for enabling an analysis of measurement accuracy, but also for determining the best slice thickness/interval to employ in clinical practice, with consideration for the stability (reproducibility) of the density measurement.

Here we describe an example of the clinical usefulness of the results such as quantitative analyses of Figs. 8 and 9 and qualitative analysis of Fig. 10. We assume that a spherical‐shaped solid lung nodule in a patient grows from 2 to 4 mm diameter while maintaining a constant density (‐400 HU); these assumptions are the same as those used in the simulations of this study. When performing a progressive observation of nodule growth by HRCT images, there is one possible case where the nodule density could be observed to change from the density of $C_{2\;\text{mm}}$ to the density of $A_{4\;\text{mm}}$, as indicated in Fig. 10. That is, the nodule density is observed to increase rapidly. In (Fig. 9f), the increase in density is estimated to be approximately 300 HU (from ‐653 of $C_{2\;\text{mm}}$ to ‐349 HU of $A_{4\;\text{mm}}$), regardless of the constant density. This misreading of a rapid density increase may be one of the factors contributing to overdiagnosis.^21^, ^23^ The results shown in Figs. 8–10 may also be useful for explaining the mechanisms of such clinical misreadings. In addition, we demonstrated that the overlap image reconstruction could be useful for avoiding this observation of rapid density increase. For example, by employing the overlap reconstruction with a slice interval of 50% of the slice thickness, the ${\text{CT}}_{\text{min}}$ of a 2 mm diameter nodule was increased from ‐653 ($C_{2\;\text{mm}}$) to ‐537 HU ($B_{2\;\text{mm}}$), as indicated in (Fig. 9f), leading to a reduction in the rapid density increase observed when the nodule grew from 2 to 4 mm. This is also observed qualitatively in Fig. 10. The results shown in Figs. 8 and 9 and accompanied by Fig. 10 could reveal clinical misreadings and lead to more accurate and precise density measurement. They may also be of value in terms of determining the optimum use of scan and reconstruction parameters, such as image reconstruction kernels and slice thickness/intervals (overlap reconstructions).

This study has some limitations. First, the simulated nodules were calculated from the object functions determined as ideal spheres with constant CT values. However, a real nodule has a complex shape with heterogeneous density. In addition, nodules could be near other anatomical structures. Numerous simulations under various conditions may be necessary, while our proposed approach of computing nodules and of adding them into clinical CT images can achieve those simulations effectively. Secondly, we did not consider noise and artifact components in the computations of nodules. The precision of nodule density measurement may be more affected by noise than by factors used in the present study, such as the offset of the nodule center relative to the voxel center. For example, normally, a 2 mm thick image would have lower noise than a 1 mm thick image. Therefore, the fluctuation in nodule density measurement may actually be lower at a 2 mm slice thickness; this consideration would be different from the results indicated in Fig. 8. Furthermore, when scanning actual patients, the measured nodule density may be affected by the beam hardening artifact. To investigate the effects of noise and artifact following the present basic study, it is necessary in future to perform the accuracy analysis, such as indicated in Figs. 8 and 9, with images that were obtained by adding simulated nodules into patient's images including actual noise and artifacts.

## V. CONCLUSIONS

Our proposed method of PSF‐based image simulation accompanied with resampling enables a quantitative analysis of the accuracy and precision of lung nodule density evaluations. Furthermore, the method is generalizable and can be applied to any 3D structures, enabling an accuracy analysis of clinical evaluations of various object densities and sizes.

## ACKNOWLEDGMENTS

This study was supported in part by a Grant‐in‐Aid for Cancer Research (19–25) from the Ministry of Health, Labor and Welfare, Japan and by a Grant‐in‐Aid for Scientific Research (C) (23602005). This research was also supported by a joint study undertaken between Niigata University and Fujitsu Limited.
